# An Allograft Glioma Model Reveals the Dependence of Aquaporin-4 Expression on the Brain Microenvironment

**DOI:** 10.1371/journal.pone.0036555

**Published:** 2012-05-09

**Authors:** Susan Noell, Rainer Ritz, Karen Wolburg-Buchholz, Hartwig Wolburg, Petra Fallier-Becker

**Affiliations:** 1 Department of Neurosurgery, University of Tübingen, Tübingen, Germany; 2 Institute of Pathology and Neuropathology, University of Tübingen, Tübingen, Germany; The University of Chicago, United States of America

## Abstract

Aquaporin-4 (AQP4), the main water channel of the brain, is highly expressed in animal glioma and human glioblastoma *in situ*. In contrast, most cultivated glioma cell lines don’t express AQP4, and primary cell cultures of human glioblastoma lose it during the first passages. Accordingly, in C6 cells and RG2 cells, two glioma cell lines of the rat, and in SMA mouse glioma cell lines, we found no AQP4 expression. We confirmed an AQP4 loss in primary human glioblastoma cell cultures after a few passages. RG-2 glioma cells if grafted into the brain developed AQP4 expression. This led us consider the possibility of AQP4 expression depends on brain microenvironment. In previous studies, we observed that the typical morphological conformation of AQP4 as orthogonal arrays of particles (OAP) depended on the extracellular matrix component agrin. In this study, we showed for the first time implanted AQP4 negative glioma cells in animal brain or flank to express AQP4 specifically in the intracerebral gliomas but neither in the extracranial nor in the flank gliomas. AQP4 expression in intracerebral gliomas went along with an OAP loss, compared to normal brain tissue. AQP4 staining *in vivo* normally is polarized in the astrocytic endfoot membranes at the *glia limitans superficialis and perivascularis*, but in C6 and RG2 tumors the AQP4 staining is redistributed over the whole glioma cell as in human glioblastoma. In contrast, primary rat or mouse astrocytes in culture did not lose their ability to express AQP4, and they were able to form few OAPs.

## Introduction

Aquaporin-4 (AQP4) is the predominant water channel in the brain and it is mainly expressed by astrocytes and ependymal cells. AQP4 exists in different isoforms [1]. The two most important and best studied AQP4 isoforms are M23 and the 22 amino acids longer isoform M1. Both isoforms exhibit different water transport capacities [2]. The peculiarity of AQP4 is its morphological appearance. AQP4 is characterized by the freeze fracturing technique revealing orthogonal arrays of particles (OAP; for a recent review, see [3]). In astrocytes, they were first described in 1973 by Dermietzel [4]. *In vitro* experiments showed that M1 transfected cells only form small or no OAPs in the cell membrane, whereas M23 transfected cells were capable to form huge lattices of OAPs. A cell which was transfected with both AQP4 isoforms showed typical OAPs which were sized as normal astrocytes in healthy brain or *in vitro* [5].

Previously, we showed that agrin was significantly involved in the formation of OAPs [6]–[8]. Agrin, which was discovered by McMahan [9], is a heparansulfate proteoglycan and a component of the extracellular matrix (ECM; [10]). This proteoglycan is known for its ability to cluster acetylcholine receptors at the neuromuscular endplate [9], [11]. Barber and Lieth [12] described the importance of agrin in the central nervous system (CNS) for the integrity of the blood-brain barrier (BBB) showing that during chick and rat brain development agrin accumulated on brain micro vessels by the time the vasculature became impermeable.

Under physiological conditions, AQP4 containing OAPs are highly polarized on the perivascular and superficial membranes of astrocytic endfoot processes, whereas parenchymal membranes contain less OAPs [13]. This points to the critical function of water transport across the BBB and brain-cerebrospinal fluid interfaces [14]. Under pathological conditions, this polarization is lost. In glioblastoma, the topology of the cellular constituents has completely changed: the glioma cells do no more form typical endfeet, and the perivasculare space has tremendously increased. Perhaps as a reaction on this, AQP4 is upregulated [15] and redistributed over the whole tumor cell surface [16]. This redistribution of AQP4 directly corresponds to loss of the water channel-related polarity leading to water movements not only between blood and glia but also between glia and brain parenchyma. Here, the water accumulates and decisively contributes to an increased edematous intracranial pressure with concomitant BBB breakdown. This is known not only in brain tumors, but also in encephalomyelitis, stroke, trauma, and other brain diseases [15], [17]–[22]. In order to investigate edema formation, commonly *in vitro* as well as *in vivo* models are used. Frequently, cell lines or primary cell cultures from glioblastoma are employed to measure the cell volume regulation [2], [6], [23], [24] but many glioma cell lines do not even express the water channel proteins aquaporin 1, 4, and 5, which are typical for glioblastomas. Moreover, the majority of freshly isolated glioma cells do not express any of these water channels *in vitro* in primary cell cultures. However, in the glioma tissue - from which the cells were isolated – AQP 1, 4, and 5 were detected [25].

*In vivo* edema formation is studied in animal models after generating tumors by implantation of glioma cells into the brain [26], [27] or flank [28].

From previous studies it is known that the ECM has a large influence on the constitution and distribution of the water channel protein AQP4 [7]–[9]. In an orthotopic xenograft model for example tumor cells have been shown to change both basal lamina components and distribution and expression of AQP4 [29].

The aim of the present study was to find out whether glioma cell lines which contain no AQP4 are capable of expressing this water channel after implantation into brain or flank. This would give a hint to the significant effect of the brain microenvironment on the expression and distribution of AQP4.

## Results

### Immunohisto- and Immunocytochemical Staining

**Figure 1:**
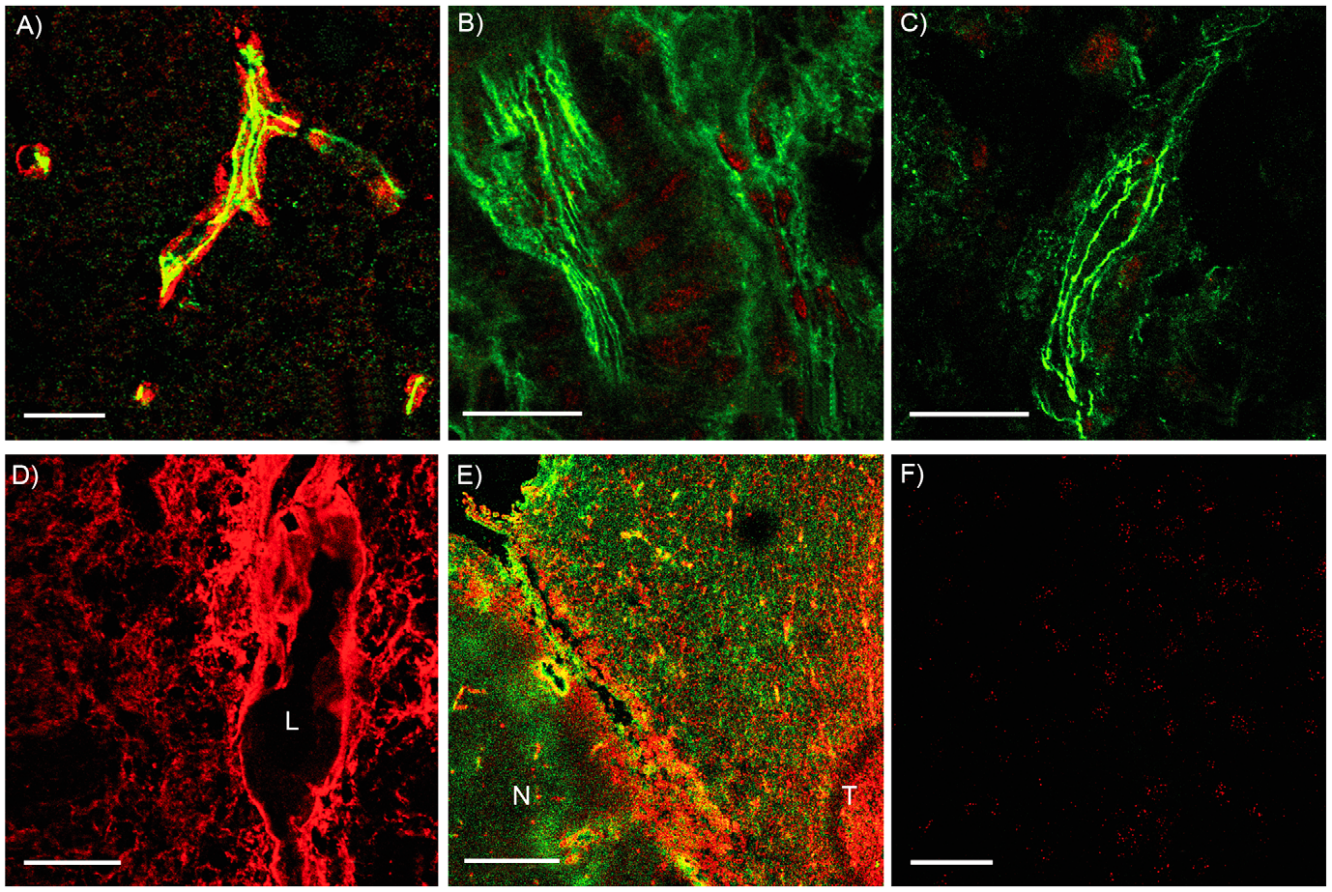
Immunoreactivity against AQP4. (A) Rat control brain, AQP4 (red) is restricted to astrocytic endfeet contacting the blood vessel (green ZO-1). (B) Extracranial implanted C6-tumor, no AQP4 could be detected, green ZO-1. (C) C6-tumor implanted into the flank, no AQP4 could be detected, green ZO-1. (D) Intracerebral implanted C6-tumor, immunofluorescence (red) shows an intensive staining for AQP4 (L: blood vessel lumen). (E) Right side: Intracerebral implanted RG-2-tumor (T); AQP4 (red) is present in astrocytes all over the tumor tissue and stronger fluorescence in the reactive astrocytes, compared to the healthy part of the brain (N), GFAP (green). Left side: healthy part of the brain (N), AQP4 (red) is restricted to astrocytic membranes contacting the blood vessels. (F) No AQP4 could be detected in C6 cell cultures. Scale bars each 20 µm.

Immunohisto- and immunocytochemical staining of AQP4 was performed on human and rat tissue as well as on cell cultures. In rat control brain, astrocytic endfeet were stained against AQP4 (red), and the vascular endothelial cell TJs against ZO-1 (green, fig. 1 A). The parenchymal tissue was weakly stained against AQP4. In contrast, the extracranial C6 tumor as well as the C6 flank tumor did not stain for AQP4 (fig. 1 B and C). Implanted C6 and RG-2 cells into the brain were forming intracerebral tumors (fig. 1D, E, respectively) and were able to express AQP4, but in contrast to normal brain the AQP4 fluorescence was redistributed and not restricted to blood vessel-associated endfeet. C6- and RG-2 cells, if cultured *in vitro* were immuno-negative for AQP4 (fig. 1 F).

**Figure 2:**
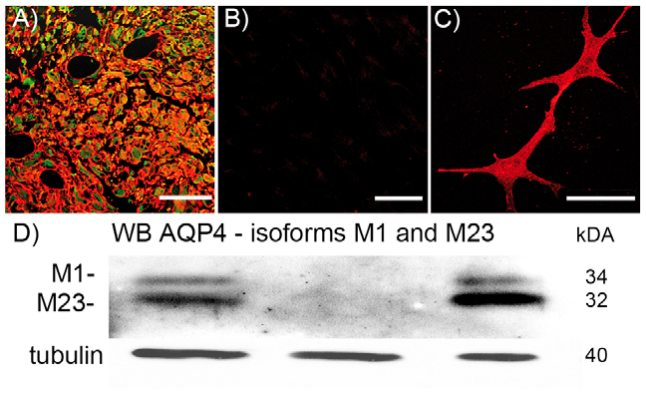
Immunoreactivity against AQP4 (red) and GFAP (green). (A) In human glioblastoma tissue AQP4 (red) shows an intensive staining whereas in primary cell culture of this glioblastoma AQP4 could not be detected (B). (C) Primary cell culture of astrocytes stained for AQP4. (D) Immunoblot against AQP4; the lower band (32 kDA) represents the AQP4 isoform M23 and the upper band (34 kDA) the isoform M1. Tubulin was used as loading control for these samples (40 kDA). The western blot is positive for AQP4 in the glioblastoma tissue (left lane), whereas the primary glioma cell cultures were negative (middle). Primary mouse astrocytes are positive for AQP4 (right lane). The M23-AQP4 isoform always shows a stronger band than the M1 isoform.

Comparable results were achieved by investigating human glioblastoma tissues and by isolating these tumor cells (fig. 2). In glioblastoma tissues, AQP4 was found in membranes surrounding the whole glioma cells (fig. 2 A). In freshly isolated cells in primary culture, no AQP4 fluorescence was detected (fig. 2B). In contrast, primary cultures of freshly isolated astrocytes of healthy mouse or rat brain revealed a strong fluorescence of AQP4 (fig. 2C).

These results were underlined by studies with mice. Cultured SMA cells did not stain for AQP4 but when implanted into mouse brains, SMA gliomas became immunoreactive for AQP4 (data not shown).

### Western Blot

**Figure 3:**
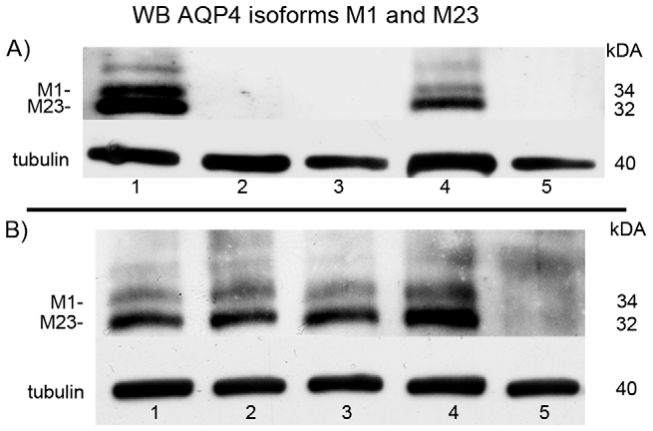
Western blot against AQP4. (A) C6-system: the lower band (32 kDA) represents the AQP4 isoform M23 and the upper band (34 kDA) the isoform M1. Rat control brain (lane 1), C6 cell culture (lane 2), extracranial implanted C6 cells (lane 3), intracerebral implanted C6 cells (lane 4) and C6 cells implanted into the rat flank (lane 5). Tubulin was used as loading control for these samples (40 kDA). (B) RG-2- system: Control rat brains (lane 1 and 3) and Intracerebral RG-2 cell tumors (lane 2 and 4) are positive for AQP4. The RG-2 cell culture (lane 5) is negative. The M23 AQP4 isoform always shows a stronger band than the M1 isoform. Tubulin was used as loading control for these samples (40 kDA).

Western blot analyses of AQP4 showed two bands referring to different isoforms. The AQP4 M23- and M1- isoforms are normally expressed in rat brain (fig. 3A lane 1, 3B lane 1 and 3). In contrast, no AQP4 bands could be detected in C6 cell cultures (fig. 3A lane 2), but if implanted into the rat brain, these cells regained the ability to express the AQP4 M23- and M1- isoforms again (fig. 3A lane 4). In contrast, no AQP4 expression could be detected in C6 cells implanted extracranially or into the flank (fig. 3A lane 3 and 5). Therefore, C6 cells seemed to be unable to express AQP4 outside the brain.

Another established glioma model of rats, the RG-2 system, showed similar results. Normal rat brain and the intracerebrally implanted RG-2 tumor expressed AQP4 (fig. 3B lane 1, 3 and 2, 4, respectively), whereas RG-2 cells in culture did not (fig. 3B lane 5). Tissue from glioblastoma patients contained AQP4 (fig. 2D lane 1) whereas it was lost in primary cell cultures isolated from these tumors, after one or more passages (fig. 2D lane 2). In contrast, primary astrocyte cultures from healthy mouse brain expressed AQP4 *in vitro* (fig. 2D lane 3). To summarize the western blot results, only in the rat control brain and in the intracerebrally implanted C6 cell tumor AQP4-bands could be detected showing always a stronger M23-band compared to the AQP4 -M1 isoform.

### RT-PCR

**Figure 4:**
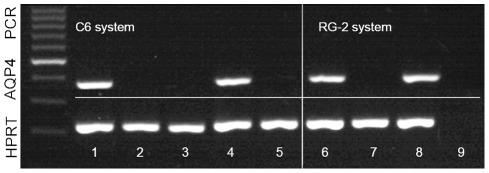
Representative RT-PCR on an agarose gel showing bands corresponding to AQP4 mRNA and the internal standard HPRT. AQP4 mRNA is only expressed in normal rat brain (lane 1 and 6) and intracerebral implanted C6 and RG-2 cell tumor (lane 4 and 8) compared to the C6 and RG-2 cell culture (lane 2 and 7) as well as C6 implanted extracranial and flank tumor (lane 3 and 5). Lane 9 shows the negative H_2_O control.

PCR analyses showed an expression of AQP4 m-RNA in normal rat brain and in the intracerebrally implanted C6 tumor (fig. 4, lane 1 and 4), but not in the implanted C6 extracranial and flank tumor (fig. 4, lane 3 and 5) and not in the C6 cell culture (fig.4 lane 2). We found similar results in the RG-2 system: RG-2 cells did not express AQP4 (fig. 4, lane 7), but intracerebrally implanted RG-2 cells showed an AQP4 expression as well as the control rat brain (fig. 4, lane 8 and 6, respectively). HPRT was used as housekeeping gene. Fig. 4, lane 9 is the master mix control with H2O instead of a sample probe.

### Freeze Fracture

**Figure 5:**
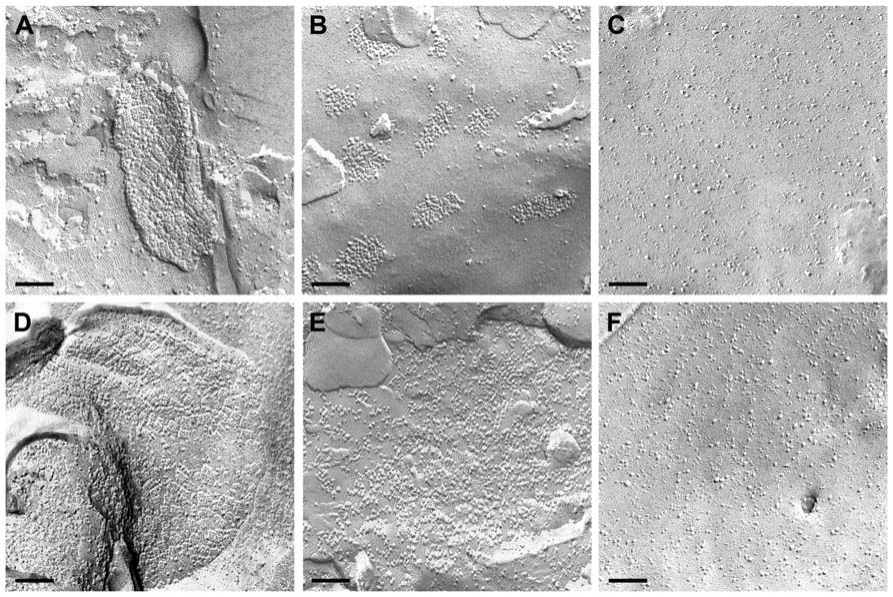
Freeze fracture analysis. (A) Normal human brain tissue and rat brain tissue (D) showing astrocytic endfoot membranes studded with OAPs containing AQP4. (B) Altered morphology of clustered OAPs in human glioblastoma tissue. (C) Primary cell culture membranes of human glioblastoma are devoid of OAPs as well as intracerebral RG-2 tumor (E), and the RG-2 cell line (F). Scale bars 100 nm.

**Figure 6:**
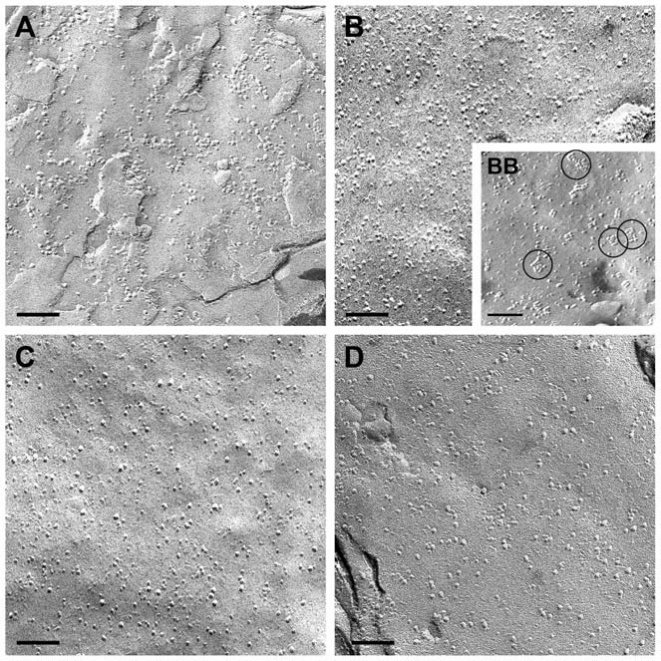
Freeze fracture analysis of the C6 system. (A) C6 implanted intracerebral tumor, (B), C6 cell culture. C6 cells implanted extracranial (C) and flank tumor (D). There are no OAPs. In contrast (BB) shows a freeze fracture replica of primary astrocytes in culture revealing a few OAPs (encircled). Scale bars 100 nm.

Freeze fracture replicas were produced to investigate the morphology of the glial cell membranes. As expected and known from numerous previous studies, the normal healthy human brain (fig. 5A) and rat brain tissue (fig. 5 D) showed astrocytic endfoot membranes studded with OAPs. In the C6 (fig. 6 A) and RG-2 (fig. 5E) intracerebral tumors, the membranes did not show typical OAPs, although they were positive for AQP4 at the protein - (fig. 3) and RNA - level (fig. 4). In human glioblastoma tissues, we were able to demonstrate altered clusters of OAPs (fig. 5B). In some clusters, the OAP structure could be recognized, while in others were not. The membranes in intracerebral C6 and RG-2 tumors did not reveal any OAPs (fig. 5E and 6A) although these cells expressed the AQP4 protein (fig. 3A lane 4, fig. 3B lane 4). C6 tumor tissue of the flank (fig. 6 D) and in extracranial position (fig. 6C) expressed no AQP4 and therefore did not form OAPs. In cultured primary glioblastoma cells, RG2 and C6 cells no AQP4 could be detected and accordingly no OAPs were found (fig. 5C, 5F and 6B). In contrast, primary astrocytes in culture were able to form OAPs *in vitro* (fig. 6BB).

## Discussion

The results of this study indicate that AQP4 expression of grafted glioma cells depends on the surrounding microenvironment. In the orthotopic xenograft model of [29] the authors have not addressed the question of whether or not the used DBTRG cells express AQP4. They compared the expression of several molecules of the neurovascular unit, including AQP4, inside and outside the grafted glioma. Most primary cells from glioblastoma tissue as well as glioma cell lines do not express AQP4 under culture conditions. McCoy and Sontheimer [30] reported that most primary cell cultures of glioblastoma did not express the water channels AQP-1, 4 and 5, but *in vivo* they did. They also observed that a lot of glioma cell lines did not express AQP4. This difference between the *in vivo* and *in vitro* situation led us to the idea to implant different AQP4 negative glioma cells into the brain. We chose two animal models, one rat and one mouse model, to prove the RG-2 data and to extend the experiments asking the question of whether the microenvironment plays a role in both the expression of AQP4 and the formation of OAPs. We implanted AQP4-negative C6 cells into the flank and brain of Wistar rats and AQP4-negative SMA cells into the flank and brain of VmdK mice. We found AQP4 expression only in intracerebral tumors. Western blot analyses showed that these brain gliomas expressed AQP4 isoform -M1 and -M23, like normal rat brain, whereas cultivated cell lines were negative for AQP4. These data could be underlined on the RNA-level. In contrast to the implantation of cell lines into the brain, the implantation of cell lines into the flank did not result in AQP4 expression confirming that the microenvironment of the brain was essential for the expression of AQP4. In addition, extracranial tumors showed the same results as flank tumors: no AQP4 was detected.

In this context, it is important to emphasize that despite of AQP4 expression including the M1- and M23-isoforms in animal glioma, no OAP formation could be observed in freeze fracture analysis. The western blot data from control brains forming OAPs showed no differences in the expression pattern of the AQP4 isoforms M1 and M23 compared to the human and animal glioma tissues, forming no OAPs. In a series of transfection experiments Furman et al. [5] showed that M1 transfected CHO-cells formed no or only little OAPs whereas M23 expressing CHO-cells formed large OAP lattices. CHO-cells transfected with both AQP4 isoforms yielded OAPs of natural size. However, in our experiments with human and glioma tissue we did not observe OAPs inspite of the presence of both AQP4-M1 and AQP4-M23. This was completely unexpected, because expression of both AQP4 isoforms should be paralleled by the formation of OAPs. The question arises, whether transfected cell lines yield veritable results concerning the informative value of *in vivo* situations.

In glioblastoma, Saadoun et al. [15] showed an upregulation and redistribution of AQP4 [31], [32]. None of these authors applied freeze-fracturing in order to test the morphological appearance of the AQP4 water channel. A few articles exist, discussing a possible role of OAPs. These groups found a short 3_10_ helix in an extracellular loop, which mediated weak but specific interactions between AQP4 molecules in adjoining membranes [33], [34]. They concluded that AQP4 played a role in cell adhesion and suggested from crystal structure data that each AQP4 tetramer interacts with tetramers in the opposing membrane, which might enhance adhesion. Accordingly, reintroduction of AQP4 into AQP4-deficient glioma cell lines enhanced cell adhesion rather than cell growth, whereas AQP1 expression led to enhanced cell growth and migration [30]. Zhang and Verkman [35] provided evidence against the involvement of AQP4 in cell-cell adhesion, investigating L-cells like the Hiroaki group. They also compared primary cultured glia cells from brains of wild type versus AQP4-deficient mice concerning AQP4- dependent aggregation. They could not confirm that AQP4 was involved in cell-cell adhesion. So the OAP function is still unclear. We could show that primary astrocytes under culture conditions formed less OAPs than *in vivo*, but we observed no changes in the AQP4 protein-expression pattern. For further studies it is essential to learn more about the composition of the microenvironment, including extracellular matrix, basal lamina and other cell types in brain and flank. For a long time it is known that the composition of the extracellular matrix in the brain is completely different to that of the skin. Proteoglycans like agrin are predominant in the brain whereas in the skin collagen, laminin and fibronectins prevail. This could be an explanation for our observation that brain tumor cell lines, which were not able to express AQP4 *in vitro*, re-expressed AQP4 after implantation into the brain, but not if implanted subcutaneously into the flank. Nevertheless, implanted tumor cells in the brain did not form OAPs. In contrast, OAPs of astrocytes in healthy mammalian brains are polarized on the astrocytic endfoot membranes touching the vascular or meningeal basal lamina [3]. Recent data of Noell et al. [6], [7] suggested agrin to play a role in OAP formation. This will be examined in further studies with implanted glioma cells in flank and brain.

Isolated astrocytes in culture loose OAP-polarity and form less OAPs comparable to parenchymal membranes which have no contact to the vascular or meningeal basal lamina in the brain. These cell cultures never stop expressing AQP4 in contrast to cultured glioma cells. The reasons are still unclear.

Comparable results related to a molecular dependence of a specialized microenvironment were achieved by Pandita et al. [28] and Giannini et al. [36], who found loss of epidermal growth factor (EGF) receptors amplification in glioblastoma cells *in vitro*. Pandita et al. [28] could solve the problem by implanting cells into the flank and found an EGFR-amplification. Such subcutaneously grown tumor cells from the flank were implanted orthogradely into the brain. These cells maintained EGFR and revealed a brain invasive phenotype [36].

Accordingly, in the present study we could show that AQP4 negative glioma cell cultures are capable of re-expressing AQP4 *in vivo* after orthotopic implantation. Healthy brain microenvironment seemed to be responsible for the re-expression of AQP4 protein but without OAP formation.

## Materials and Methods

### Cell Culture

Rat glioma C6 (C6BU-1) cells (American Type Culture Collection, Rockville, MD, USA [37] and RG-2 cells (Rat glioma cells, [38] kindly provided by W. Kugler, Göttingen, Germany) were cultivated in RPMI medium containing 10% fetal bovine serum and penicillin (10.000 units/ml; Lonza, Cologne, Germany), streptomycin (10.000 µg/ml; Lonza, Cologne, Germany). SMA cells (mouse glioma cells [39] kindly provided by U. Naumann, Hertie Institute, Tuebingen, Germany) were cultivated in DMEM containing 10% fetal bovine serum and penicillin (10.000 units/ml; Cologne, Germany), streptomycin (10.000 µg/ml; Lonza, Cologne, Germany). All cells were cultured until they reached confluence. After washing with PBS (Invitrogen, Karlsruhe, Germany) cells were prepared for freeze fracturing, immunocytochemistry, PCR and implantation.

### *In vivo* Implantation of C6 Cells and SMA Cells

Animal care and all experimental protocols were conformed to the animal ethics committee guidelines of the University of Tuebingen and the German legislation, regulating the use of animals in research. 6 Wistar rats (Charles River, Sulzfeld, Germany) weighing 200–250 g underwent implantation of glioma C6 cells (105) in 2 µl PBS. Implantation was performed using a stereotactic frame. Rats were anaesthetized using a combination of fentanyl (0.005 mg/kg bodyweight(bw)), midazolam (2.0 mg/kg bw) and medetomidine (0.15 mg/kg bw) by intraperitoneal injection. A scalp incision of 10 mm length was made over the frontal-parietal area. A hole was made 2 mm lateral to the sagittal suture and 2 mm in front of the coronar suture using a microsurgical drill. Rats were fixed in the frame and the cell suspension was implanted slowly under the dura mater of the brain in 2 mm depth. The hole was closed using bone wax and the scalp was sewed. In addition 105 cells were implanted in the right flank of the rats via subcutaneous injection. After the implantation, rats got antidote and painkiller once. Rats were observed 10 to 14 days, then anesthetized and decapitated. The whole brain, in some cases extracranial tumor and the tumor of the right flank was frozen in tissue tekR (Sakura, USA) for immunohistochemical analysis and PCR.

Six VmDK-mice (kindly provided by U. Naumann, Hertie Institute, Tuebingen, Germany) underwent a similar procedure as described above for Wistar rats. The difference was the implanted cell type, using SMA cells instead of C6 cells.

### RG-2 Tumors

RG-2 tumor implantations in 4 rats were performed in childreǹs hospital (University of Göttingen, Germany) by the group of Prof. M. Lakomek.

### Human Glioblastoma Tissue

We investigated tumor samples from patients with primary glioblastomas treated in the Department of Neurosurgery Tübingen. As control, brain tissue from one patient suffering from a meningioma was examined which was removed in order to facilitate the approach for tumor resection.

**Table 1:** List of primer sequences used in this study.

Target mRNA (rat)	Primer Sequences (5′ ->3′)	Product size (bp)	Annealing (°C)
AQP4		367	62
s	CAA AGG GGT CTG GAC TCA AGC		
as	TGG TGA CTC CCA ATC CTC CAA C		
HPRT		218	60
S	GCT TTT CCC GCG AGC CGA CCG GT		
AS	AGG GCC ACA ATG TGA TGG CCT C		

### Immunohisto- and Immunocytochemistry

This method was performed on 7 µm slices of the frozen tissues and on C6, RG-2 or SMA cells seeded and grown on coverslips. The following primary antibodies were used: anti-aquaporin-4 (1∶100, rabbit polyclonal, Santa Cruz, Heidelberg, Germany), anti-GFAP 1∶100 (mouse monoclonal, Dako, Hamburg, Germany), and anti-ZO-1 (1∶100 mouse monoclonal, Invitrogen, Darmstadt, Germany). The secondary goat anti-mouse and anti-rabbit antibodies labelled with cyanin-derivative dye Cy3 or Cy2 were purchased from Dianova (Hamburg, Germany). For controls, the primary antibody was omitted. Fluorescence was visualized with a ZEISS LSM 510 META confocal laser scanning microscope (ZEISS Oberkochen, Gemany).

### Western Blotting

Confluent C6, RG-2 and SMA cells or slices of frozen tissues (extracranial tumor, intracerebral tumor, normal rat brain and tumor of the flank) were lysed and prepared for western blotting as described by Neely et al. [40]. Briefly, cells or tissue were lysed with Laemmli-buffer, and protein was measured using the method of Bradford [41]. Five µg of total protein of each sample was used for electrophoreses with 12.5% SDS-PAGE gel. The samples were blotted on a nitrocellulose membrane and stained with an antibody against AQP4 (Santa Cruz, Heidelberg, Germany) and a secondary antibody labeled with horseradish peroxidase (Sigma, Taufkirchen, Germany).

### Reverse Transcriptase (RT)-PCR

RT-PCR analysis for AQP4 and hypoxanthine guanine phosphoribosyl transferase (HPRT) mRNAs was performed as described previously [42]. The HPRT gene has been reported as a constitutively expressed house-keeping gene [43]. Total RNA was isolated using the peq Gold RNApure extraction kit (Peqlab, Erlangen, Germany) according to the manufacture’s protocol. The cDNA was synthesized from 1 µg of each RNA, 1 µl dNTP (0.8 nM), 1 µl (MMLV) reverse transcriptase, 5 µl 5x-buffer and 1.5 µL hexanucleotide (10 pmol/µL; all reagents from Invitrogen, Karlsruhe, Germany) for 1 h at 37°C followed by enzyme inactivation for 5 min at 95°C. PCR was conducted with 1.5 µL probe of RT reaction, 0.5 µl sense and antisense primers, 1 µl (5 mM) dNTP, 2.5 µl 10x PCR-buffer, 0.75 µl (2.5 mM) MgCl2, and 0.3 µl AmpliTaq polymerase (Applied Biosystems, Fosters City, California, USA). PCR conditions were: 32 cycles (AQP4- and HPRT- primers) denaturation for 30 sec at 95°C, annealing for 30 sec at 62°C (HPRT, AQP4 primers) extension for 30 sec at 72°C, followed by a final elongation step at 72°C for 5 min Professional BASIC Cycler (Biometra, Göttingen, Germany). Concurrent RTPCR amplification of HPRT was carried out as an internal control for variations in the efficiencies of RNA isolation and RT. The primer sequences are shown in Table 1.

The PCR products were separated by electrophoresis on a 1.5% agarose gel, stained with FastRed and analysed with a UV transilluminator. RT-PCRs were confirmed in replicates. Prior to the semiquantitative analysis, we determined that 32 PCR cycles were well within the linear detection range.

### Freeze-fracture Experiments

Monolayers of cultured cells or tissue were fixed with 2.5% glutaraldehyde in 0.1 M cacodylate buffer (pH 7.4) for 2 h at room temperature. The specimens were then cryoprotected for freeze-fracturing in 30% glycerol and quick-frozen in nitrogen-slush (−210°C). Subsequently, they were fractured in a Balzer’s freeze-fracture device (BAF400D; Balzers, Liechtenstein) at 5×10–6 mbar and −150°C. The fracture faces were shadowed with platinum/carbon (3 nm, 45°) for contrast and carbon (30 nm, 90°) for stabilization of the replica. After removal of the cell material in 12% sodium hypochlorite, the replicas were rinsed in double-distilled water several times and mounted on Pioloform-coated copper grids. The replicas were observed using a Zeiss EM10 electron microscope (Zeiss, Oberkochen, Germany).
